# Comparing the frequency of isotretinoin-induced hair loss at <0.5-mg/kg/d versus ≥0.5-mg/kg/d dosing in acne patients: A systematic review

**DOI:** 10.1016/j.jdin.2022.01.002

**Published:** 2022-02-10

**Authors:** Yuliya Lytvyn, Katherine McDonald, Asfandyar Mufti, Jennifer Beecker

**Affiliations:** a Faculty of Medicine, University of Toronto, Toronto, Ontario, Canada; b Department of Dermatology, University of Toronto, Toronto, Ontario, Canada; c Division of Dermatology, University of Ottawa, Ottawa, Ontario, Canada; d The Ottawa Hospital, Ottawa, Ontario, Canada; e Ottawa Research Institute, Ottawa, Ontario, Canada; f Probity Medical Research Inc., Waterloo, Ontario, Canada

**Keywords:** Accutane, alopecia, Clarus, Epuris, hair loss, hair shedding, hair thinning, high dose, isotretinoin, low dose, telogen effluvium

## Abstract

Over 1 million isotretinoin prescriptions are authorized in the United States per year. An insight into the frequency, dose dependency, timing, and reversibility of hair loss associated with isotretinoin treatment for acne vulgaris could help guide dosing regimens and patient counseling. The objective of this systematic review was to assess the frequency of hair loss in patients with acne vulgaris on <0.5 mg/kg/d daily doses of isotretinoin versus the frequency of hair loss in patients with acne vulgaris on ≥0.5 mg/kg/d daily doses of isotretinoin.

An Embase and MEDLINE search was conducted on July 15, 2020, in accordance with the Preferred Reporting Items for Systematic Reviews and Meta-Analyses guidelines. The review focused on acne vulgaris patients. The treatment of acne vulgaris is the most common use of isotretinoin, and the population is typically younger and with fewer comorbidities.

Twenty-two studies reported hair loss with oral isotretinoin treatment. A frequency analysis suggested that patients with acne vulgaris on <0.5 mg/kg/d of isotretinoin experienced hair loss at a frequency of 3.2% (n = 18/565) compared with those on ≥0.5 mg/kg/d, who experienced hair loss at a frequency of 5.7% (n = 192/3375). Inferential statistics were not possible.

Physicians should consider counseling patients about the risk of telogen effluvium prior to drug initiation, as is commonly done for other side effects. The potential trend of increased hair loss frequency at a higher daily dosing warrants further investigation using higher-quality research.

**Capsule Summary**

• There are limited published data on isotretinoin-induced hair loss. This systematic review compiles available data suggesting a frequency between 3.2% and 5.7%. Further studies are required for the analysis of dose dependency.
• Physicians should consider discussing the possibility of telogen effluvium with patients.

## Introduction

Acne vulgaris is a common disease that affects up to 80% of teenagers,^1^, ^2^, ^3^, ^4^, ^5^ half of whom continue to experience acne in adulthood.^1^^,^^2^^,^^5^, ^6^, ^7^, ^8^, ^9^ The most clinically effective first-line therapy for nodular or inflammatory acne vulgaris is isotretinoin, with >1 million prescriptions per year in the United States.^5^^,^^10^ Given the volume of patients on this medication, the side effects that occur at seemingly lower frequencies represent a large cohort of patients. Although isotretinoin has a significant benefit and improves the quality of life, it has a broad side effect profile because of the expression of retinoic acid receptors throughout the body.^11^ The most common side effects are related to dry skin and mucocutaneous membranes.^11^

Hair loss in the form of telogen effluvium is a reported side effect of isotretinoin that can lead to treatment discontinuation. Although its mechanisms are unclear, retinoids are thought to arrest the onset of the anagen phase of the hair cycle and impair the anchoring of hair during the telogen phase, ultimately increasing hair shedding.^11^^,^^12^

Current product monographs of commonly used isotretinoin formulations in North America (Clarus, Epuris, and Accutane) list hair loss as a rare side effect and warn that it may persist after treatment is completed.^13^, ^14^, ^15^ The Clarus monograph reports that 13% of patients experience hair loss (presumably, these data are from pivotal clinical trials because other references are not provided) but does not describe the doses at which hair loss is observed.^13^ In a recent review of postmarketing adverse events, hair loss was reported in 932 cases (9% of all dermatologic adverse events), 62.7% of which occurred in patients between 15 and 30 years of age.^16^

With the exception of teratogenicity, the incidence and severity of side effects associated with isotretinoin are generally dependent on the dose and reversible with drug discontinuation.^11^^,^^17^^,^^18^ In the past, a typical treatment regimen was started at 0.5 mg/kg/d and increased to 1.0 mg/kg/d to reach a cumulative dose of 120 to 150 mg/kg.^18^, ^19^, ^20^ This approach was modified after studies demonstrated equal response rates and less adverse effects with lower cumulative doses.^21^, ^22^, ^23^, ^24^, ^25^, ^26^, ^27^, ^28^, ^29^, ^30^, ^31^, ^32^, ^33^ This was contradicted by other studies that suggested that acne relapse rates are greater with lower cumulative doses.^34^, ^35^, ^36^ The 2018 Global Alliance to Improve Outcomes in Acne reviewed the conflicting data and determined that to date, no high-quality clinical trials have defined a total cumulative dose that maintains remission.^37^ The most recent recommendations indicate that the appropriate evidence-based approach is to continue treatment for over 2 months after complete acne resolution.^37^, ^38^, ^39^, ^40^

Dermatologists may adjust the isotretinoin dose throughout the course of the treatment to balance the response to therapy, with more common side effects such as dryness. However, it is currently unknown whether dose reduction is effective in managing isotretinoin-induced hair loss. The exact frequency and associated dose dependency of hair loss with the use of isotretinoin remain unclear. Therefore, the primary objective of this systematic review was to assess the frequency of hair loss in patients with acne vulgaris on daily oral doses of <0.5 mg/kg/d versus ≥0.5 mg/kg/d of isotretinoin. The goal was to assess whether there are sufficient data to provide evidence-based guidelines for dermatologists prescribing isotretinoin to patients with hair loss concerns, thus preventing the avoidance or discontinuation of this medication.

## Methods

The Preferred Reporting Items for Systematic Reviews and Meta-Analyses guidelines were followed for this systematic review.^41^

### Search strategy

Articles published between January 2005 and July 2020 were retrieved from Embase and MEDLINE electronic databases in Ovid on July 15, 2020 (Supplementary File 1, available via Mendeley at https://data.mendeley.com/datasets/hrhkctyppr/1). The titles, abstracts, and full texts of the retrieved articles were independently screened by 2 reviewers (Drs Lytvyn and McDonald), and a third reviewer (Dr Mufti) resolved any disagreements. The reference lists of the relevant articles were manually searched by 1 reviewer (Dr Lytvyn).

### Inclusion criteria and study design

Original articles written in English were included if they involved the following: (1) human patients, (2) the use of isotretinoin to treat acne vulgaris, (3) hair loss outcomes, and (4) an observational or experimental study design.

Many of the available studies subdivided the data into doses of ≥0.5 mg/kg/d or those <0.5 mg/kg/d. To extract these data and reference a clinically meaningful dose (the lower end of the recommended range),^18^, ^19^, ^20^ a threshold value of 0.5 mg/kg/d was maintained in this review.

### Data abstraction

Two reviewers (Drs Lytvyn and McDonald) independently extracted data on study design, patient demographics and medical history, isotretinoin treatment (daily dose, cumulative dose, and duration), and reported hair loss. The isotretinoin doses reported in this systematic review were the target daily doses achieved and were maintained after titrating up the dose over a 1- to 2-month period. When possible, the isotretinoin dosing was converted to daily weight-based dosing for a straightforward comparison.

If available, information for the following 3 outcomes was extracted:

1. The number of patients experiencing hair loss, reported as the percentage of all patients treated in the study.
2. Time to hair loss onset: time between the first isotretinoin dose and the first report of hair loss.
3. Reversibility of hair loss associated with isotretinoin.

### Analysis

A descriptive analysis was performed because of considerable heterogeneity in the reported data and study designs of the included articles. To illustrate the general trends, the overall frequency of hair loss was calculated for the 2 groups: patients taking <0.5 mg/kg/d versus those taking ≥0.5 mg/kg/d of oral isotretinoin.

### Quality assessment

The quality of evidence was assessed independently in duplicate by 2 reviewers (Drs Lytvyn and McDonald) using the Oxford Centre for Evidence-Based Medicine, 2011, levels of evidence.^42^

## Results

### Study selection

The study focused on isotretinoin use in acne patients because this is the most common use of the drug and the patients are typically younger and with fewer comorbidities contributing to hair loss.

**Fig 1:**
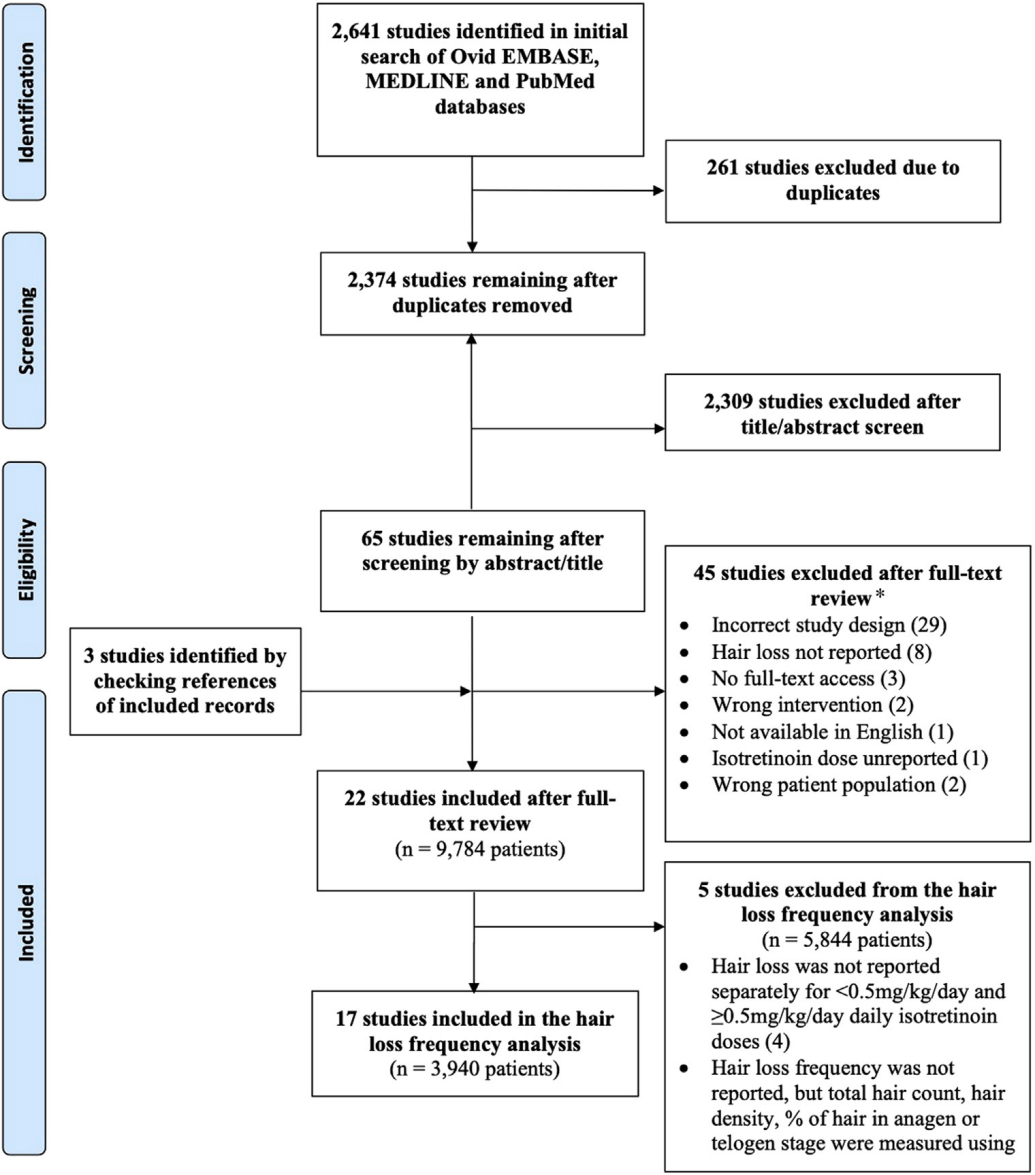
Flow diagram of literature screening using the Preferred Reporting Items for Systematic Reviews and Meta-Analyses guidelines. Figure adapted from http://prisma-statement.org. ∗Original articles written in English were included if they involved study population of interest (ie, human patients), involved the use of isotretinoin, or had an observational (ie, case reports, case series, and cross-sectional or cohort studies) or experimental (ie, randomized controlled trials) study design.

Twenty-two studies (n = 9783 patients) were identified (Fig 1, Table I),^11^^,^^22^^,^^43^, ^44^, ^45^, ^46^, ^47^, ^48^, ^49^, ^50^, ^51^, ^52^, ^53^, ^54^, ^55^, ^56^, ^57^, ^58^, ^59^, ^60^, ^61^, ^62^ 17 of which (n = 3940) were included in the frequency analysis summary (Table II). One of the studies excluded from frequency analysis did not report the frequency of hair loss and instead quantified the total hair count, hair density, and percentage of hair in the anagen or telogen phase. The remaining 4 studies that were not included in the frequency analysis did not discuss the dose at which hair loss occurred.

**Table I:** Summary of reported hair loss with <0.5 mg/kg/d and ≥0.5 mg/kg/d daily doses of oral isotretinoin in patients with acne vulgaris

Study characteristics			Patient demographics			Isotretinoin information	Hair loss diagnosis and prognosis					Other side effects (frequency)
Sources	Study design (evidence level)∗	Sample size (n)	Age (y) / sex	Comorbidities	Concomitant medications (dose and frequency)	Dose and duration	Number of patients reporting hair loss or thinning (%)	Time of onset	Measurement of hair loss	Reversibility (time)	Isotretinoin discontinuation due to hair loss	
**Daily dose isotretinoin of <0.5mg/kg/d vs ≥0.5mg/kg/d**∗												
Agarwal et al (2011)^22^	Prospective randomized study (1b)	27	19.2 / 59% M	Acne vulgaris (mild n = 9, moderate n = 9, severe n = 9)	Treated with azithromycin daily for the first 3 wk	WBD: 1 mg/kg/d CD: 112 mg/kg (calculated as 1 mg/kg/d × 112 d) Duration: 16 weeks (112 d)	0 (0%)	N/A	Patient-reported	N/A	N/A	Cheilitis (100%), dry skin (92.6%), dry mouth (66.7%), dry eyes (37.0%), dry nose (25.9%), rashes or facial redness (29.6%), abnormal lipid profile (7.4%), abnormal liver function tests (3.7%)
		28	19.1 / 57% M	Acne vulgaris (mild n = 9, moderate n = 10, severe n = 9)	Treated with azithromycin daily for the first 3 wk	WBD: 1 mg/kg/2 d CD: 56 mg/kg (calculated as 1 mg/kg/2 d × 112 d) Duration: 16 wk (112 d)	0 (0%)	N/A	Patient-reported	N/A	N/A	Cheilitis (92.9%), dry skin (78.6%), dry mouth (28.6%), rashes or facial redness (25.0%), abnormal lipid profile (3.5%)
		28	19.4 / 36% M	Acne vulgaris (mild n = 9, moderate n = 9, severe n = 10)	Treated with azithromycin daily for the first 3 wk	WBD: 1 mg/kg/d for 1 wk every 4 wk CD: 28 mg/kg (calculated as 1 mg/kg/4 d × 112 d) Duration: 16 wk (112 d)	0 (0%)	N/A	Patient-reported	N/A	N/A	Cheilitis (78.6%), dry skin (71.4%), dry mouth (17.9%), dry eyes (10.7%), dry nose (10.7%), rashes or facial redness (25.0%)
		29	18 / 55% M	Acne vulgaris (mild n = 10, moderate n = 10, severe n = 9)	Treated with azithromycin daily for the first 3 wk	Fixed: 20 mg/2 d WBD: NR CD: NR Duration: 16 wk (112 d)	0 (0%)	N/A	Patient-reported	N/A	N/A	Cheilitis (89.7%), dry skin (79.3%), dry mouth (20.9%), rashes or facial redness (17.2%)
Faghihi et al (2017)^43^	Prospective randomized clinical trial (1b)	36	22.9 / 13.9% M	Moderate and severe acne vulgaris	Treated with 250 mg of azithromycin daily for the first 2 wk as well as 0.25 mg of prednisolone in the first wk	WBD: 0.25 mg/kg/d CD: 45.6 mg/kg (calculated as 0.25 mg/kg/d × 182.5 d) Duration: 6 mo (182.5 d)	2 (5.6%)	Exact time NR but noted at 6 mo F/U	Dermatologist-reported	NR	0 (0%)	Itching (11.1%), dry mouth (2.8%), dry nose (16.7%), dry eyes (11.1%), spontaneous skin damage (2.8%), skin redness (2.8%), palm and sole skin scaling (2.8%), skin photosensitivity (5.6%), nail damage (5.6%), eye photosensitivity (2.8%), joint pain (5.6%), GI effects (11.1%), headache (2.8%), depression (5.6%)
		30	23.1 / 26.7% M	Moderate and severe acne vulgaris	Treated with 250 mg of azithromycin daily for the first 2 wk as well as 0.25 mg of prednisolone in the first wk	WBD: 0.5 mg/kg/d CD: 91.3 mg/kg (calculated as 0.50 mg/kg/d × 182.5 d) Duration: 6 mo (182.5 d)	7 (23.3%)	Exact time NR but noted at 6 mo F/U	Dermatologist-reported	NR	0 (0%)	Itching (20%), dry mouth (20%), dry nose (40%), repeated rhinorrhea (20%), dry eyes (20%), skin redness (13.3%), palm and sole skin scaling (3.3%), skin photosensitivity (20%), palm and sole burning (3.3%), nail damage (6.7%), poor night vision (10%), eye photosensitivity (6.7%), muscle pain (3.3%), joint pain (3.3%), GI effects (16.7%), headache (6.7%), fatigue (6.7%), depression (6.7%)
**Daily isotretinoin dose of <0.5mg/kg/d**												
De and Kanwar (2011)^44^	Preliminary, open-label, prospective, noncomparative, single-center study (1b)	66	20.4 / 61% M	Grade 3 and 4 acne vulgaris (severe and nodulocystic)	Azithromycin (500 mg/d over 3 consecutive d for 2 wk)	WBD: 0.3 mg/kg/d CD: 49.6 mg/kg Duration: Average 21 wk	2 (3.0%)	Exact time NR, noted at monthly F/Us	Patient-reported	NR	0 (0%)	Cheilitis (48.5%), xerosis (16.7%), erythema (6.1%), aphthae (3.0%), menstrual irregularities (1.5%), lipid profile abnormalities (1.5%)
Rao et al (2014)^45^	Prospective, noncomparative study (1b)	50	26.4 / 76% M	Moderate-to-severe acne vulgaris: Grade II (12%), grade III (56%), grade IV (32%) Nodulocystic acne (44%), resistant to treatment acne (40%), frequent relapses of acne (16%)	NR	WBD: 0.3-0.4 mg/kg/d CD: 27.4-36.5 mg/kg (calculated as 0.3 mg/kg/d × 91.25 d to 0.4 mg/kg/d × 91.25 d) Duration: 3 mo (91.25 d)	2 (4.0%)	Exact time NR, noted at 1 mo or 3 mo F/U	Patient-reported	NR	0 (0%)	Cheilitis (98%), Xerosis (84%), dandruff (12%), erythema of the face (8%), rough and dry hair (8%), dry mouth (6%), abnormal lipid levels (6%), abnormal liver enzyme levels (6%), rise in total cholesterol and serum triglycerides (6%), rise in liver enzymes (6%), headache (4%), vitiligo (2%)
Yap (2017)^46^	Prospective (1b)	150	26.6 / 48% M	Moderate (64.7%), severe (29.3%) and very severe (6%) acne	NR	Fixed: 10 mg/d WBD: 0.17 mg/kg/d (calculated as fixed dose 10 mg/59.0 kg mean) CD: average 98.8 mg/kg Duration: 2-3 mo	2 (1.3%)	Exact time NR, noted at biweekly F/U	Patient-reported	NR	0 (0%)	Lip dryness (100%), xerosis (36%), eczema (4%), liver enzyme elevation (3.3%), lipid increase (2.7%), mood change (0.7%)
Dhaked et al (2016)^47^	Randomized, comparative, prospective study (1b)	118	19.0 / 83% M	Moderate-to-severe acne	NR	Fixed: 20 mg/d WBD: NR CD: NR Duration: 24 wk (168 d)	7 (5.9%)	Exact time NR, noted at biweekly F/U	Patient-reported	NR	0 (0%)	Cheilitis (97.5%), dry skin (16.9%), dry mouth (2.5%), dry eyes (7.6%), dry nose (3.4%), facial erythema (2.5%), pruritus (9.3%), urticaria (3.4%), headache (1.7%), oral aphthous (0.8%), menstrual irregularities (5%), arthralgia (0.8%), myalgia (1.7%), abnormal lipid profile (3.4%), abnormal liver function tests (2.5%), forgetfulness (0.8%)
		116	18.8 / 78% M	Moderate–to-severe acne	NR	Fixed: 20 mg/2 d WBD: NR CD: NR Duration: 24 wk (168 d)	3 (2.6%)	Exact time NR, noted at biweekly F/U	Patient-reported	NR	0 (0%)	Cheilitis (95.7%), dry skin (10.3%), dry mouth (1.7%), dry eyes (1.7%), dry nose (0.9%), facial erythema (2.6%), pruritus (4.3%), urticaria (2.6%), headache (0.9%), oral aphthous (0%), menstrual irregularities (8%), myalgia (1.7%), abnormal lipid profile (0.9%), abnormal liver function tests (0.9%), pigmentation of face (0.8%), dermographism (0.8%)
**Daily isotretinoin dose of strictly ≥0.5mg/kg/d**												
Kus et al (2005)^48^	Investigator-blinded, randomized prospective study (1b)	39	21.8 / 43.9% M	No significant systemic disease	NR	WBD: 1 mg/kg/d CD: 112 mg/kg (calculated as 1 mg/kg/d × 112 d) Duration: 16 wk (112 d)	19 (48.7%)	Exact time NR, noted at 4, 8, 12, or 16 wk F/U	Patient-reported, hair loss was asked about among other side effects	NR	0 (0%)	Facial erythema (74.4%), facial dryness (82.1%), desquamation of lips (100%), body dryness (76.9%), nasal crusting (82.1%), cheilitis (79.5%), eye irritation (20.5%), epistaxis (23.1%), bruising of skin (7.7%), pyogenic granuloma (7.7%), MS symptoms (43.6%), GI symptoms (15.4%), headache (25.6%)
		36	21.3 / 51.2% M	No significant systemic disease	Vitamin E (800 IU/d)	WBD: 1 mg/kg/d CD: 112 mg/kg (calculated as 1 mg/kg/d × 112 d) Duration: 16 wk (112 d)	14 (38.9%)	Exact time NR, noted at 4, 8, 12, or 16 wk F/U	Patient-reported, hair loss was asked about among other side effects	NR	0 (0%)	Facial erythema (83.3%), facial dryness (91.7%), desquamation of lips (100%), body dryness (91.7%), nasal crusting (86.1%), cheilitis (88.9%), eye irritation (33.3%), epistaxis (27.8%), pyogenic granuloma (8.3%), MS symptoms (41.7%), GI symptoms (19.4%), headache (16.7%)
Akman et al (2007)^49^	Multicenter, controlled prospective study (1b)	22	22.7 / 36.4% M	Moderate (grade 2) to severe (grade 3-4) acne vulgaris	NR	WBD: 0.5 mg/kg/d CBD: 25.0 mg/kg Duration: First 10 d of each mo for 6 mo	0 (0%)	N/A	Patient-reported, hair loss was asked about among other side effects	N/A	N/A	Dryness in mouth (9%), dry chapped lips (73%), dry skin (59%), dry or irritated eyes (9%), pruritus (14%), rash or facial redness (5%), excessive thirst (9%)
		19	20.0 / 52.6% M	Moderate (grade 2) to severe (grade 3-4) acne vulgaris	NR	WBD: 0.5 mg/kg/d CBD: 48.8 mg/kg Duration: Each d in the first month, then the first 10 d of each mo for 5 mo	0 (0%)	N/A	Patient-reported, hair loss was asked about among other side effects	N/A	N/A	Dryness in mouth (5%), dry chapped lips (95%), dry skin (65%), dry or irritated eyes (5%), dryness of other mucosal tissues (5%), pruritus (16%), rash or facial redness (32%), peeling of fingertip skin (11%), excessive thirst (21%)
		19	20.0 / 26.3% M	Moderate (grade 2) to severe (grade 3-4) acne vulgaris	NR	WBD: 0.5 mg/kg/d CBD: 101.4 mg/kg Duration: 6 mo	0 (0%)	N/A	Patient-reported, hair loss was asked about among other side effects	N/A	N/A	Dryness in mouth (47%), dry chapped lips (100%), dry skin (78%), dry or irritated eyes (16%), dryness of other mucosal tissues (21%), pruritus (32%), rash or facial redness (47%), epistaxis (11%), peeling of fingertip skin (21%), fatigue (5%), excessive thirst (16%)
Burger et al (2014)^50^	Cross-sectional/questionnaire (2b)	57	20.2 / 57.9% M	Acne vulgaris	NR	Fixed: Average 44.2 mg/d WBD: NR CD: NR Duration: Average 6.2 mo (188.58 d)	13 (22.8%)	NR	Patient-reported	NR	NR	Dry lips (98.2%), dry skin (87.7%), initial acne flareup (63.2%), dry eyes (56.1%), epistaxis (54.4%), sunburn (52.6%), backache (43.9%), depression (42.1%), fatigue (42.1%), muscle pain (40.4%), dizziness (38.6%), headache (38.6%), slow healing wounds (38.6%), joint pain (38.6%), neck stiffness (31.6%), sudden urges to fall asleep (29.8%), constipation (29.8%), loss of appetite (24.6%), blurred vision (22.8%), anxiety (19.3%), ingrown nails (17.5%), weight loss (17.5%)
Brito et al (2010)^51^	Prospective study (1b)	150	Range: 15-32 / 52% M	Acne vulgaris	NR	WBD: 0.5-1 mg/kg/d CD: average 120 mg/kg Duration: 3.9-7.9 mo (calculated as 120 mg/kg/0.5 mg/kg/d × 0.033 mo/d to 120 mg/kg/1 mg/kg/d × 0.033 mo/d)	34 (22.7%)	Exact time NR, noted at 1 mo and at every 3 mo thereafter at F/U	Patient-reported	NR	NR	Cheilitis (94%), xeroderma (47.3%), dryness of mucosae (46.7%), palmoplantar scaling (20.7%), epistaxis (21.3%), blepharoconjunctivitis (32%), nail fragility (25.3%), pyogenic granuloma (13.3%), pyodermitis (19.3%), flareups (6%), osteomuscular pain (11.3%), headache (9.3%), hearing complaints (1.3%), tachycardia (0.7%)
Gorpelioglu et al (2010)^52^	Prospective study (1b)	40	25 / 12% M	Acne vulgaris	NR	WBD: 0.5-1 mg/kg/d CD: 45.6-91.3 mg/kg (calculated as 0.5 mg/kg/d × 91.25 d to 1 mg/kg/d × 91.25 d) Duration: At least 3 mo (91.25 d)	1 (2.5%)	Exact time NR, noted at monthly F/U	Patient-reported	NR	0 (0%)	Dry mouth (75%), epistaxis (40%), dry chapped lips (57.5%), dry skin (52.5%), dry or irritated eyes (22.5%), dryness of other mucosal tissues (20%), pruritus (17.5%), rash or facial redness (12.5%), peeling of fingertip skin (7.5%), fatigue (10%), bone or joint ache and pain (5%), muscular cramps or pain (7.5%), excessive thirst (10%)
Entezari-Maleki et al (2011)^53^	Cross-sectional (2b)	239	24.5 / 19% M	Otherwise healthy	NR	WBD: Average 0.53/kg/d CD: Average 116.7 mg/kg Duration: Average 7.5 mo	18 (7.5%)	NR	Patient-reported	NR	NR	Dry lip (90%), dry skin (40%), increased cholesterol (24.2%), increased triglyceride (22.7%), increased LDL (18.3%), dry eye (15%), increased fasting blood sugar (10.8%), skin patch (8.5%), fatigue (7.5%), blurred vision (7.5%), dry nose (6.2%), epistaxis (5.5%), nervousness (5.5%), headache (5%), back pain (4.5%), arthralgia (4%), erythema (3.8%), xerostomia (3.3%), myalgia (3%), itching (2.5%), skin sensitiveness (2.5%), dry hair (2.5%), rash (2.5%), drowsiness (2.5%), worseness of acne (2.5%), muscle weakness (2%), eye pain (1.6%), bone pain (1.6%), weight loss (1.6%), thinning of hair (1.25%), thinning of skin (1.25%), eczema (1.25%), depressed mood (1.25%), insomnia (1.25%), weakness (1.25%), gastric pain (1.2%), skin darkness (0.8%), flushing (0.8%), skin wound (0.8%), photophobia (0.8%), loss of vision at night (0.8%), menstrual dysfunction (0.8%), nausea (0.8%), abdominal pain (0.8%), constipation (0.8%), thirst (0.8%), chest pain (0.8%), GI cramping (0.8%), kidney and bladder pain (0.4%), anorexia (0.4%), weight gain (0.4%), rhinorrhea (0.4%), dyspnea (0.4%), irritation of the throat (0.4%), dream abnormality (0.4%), gum bleeding (0.4%), skin spot (0.4%), conjunctivitis (0.4%), contact lens intolerance (0.4%), contact lens intolerance (0.4%), hair fragility (0.4%), vertigo (0.4%), amnesia (0.4%)
Tahir (2011)^54^	Prospective study (1b)	250	21.3 / 44.8% M	Acne vulgaris	NR	WBD: 1 mg/kg/d CD: 112 mg/kg (calculated as 1 mg/kg/d × 112 d) Duration: 16 wk (112 d)	52 (20.8%)	Exact time NR, noted at F/Us every 4 wk	Patient-reported	NR	0 (0%)	Cheilitis (99.2%), dryness face (80.0%), erythema face (39.2%), skin dryness (33.6%), acne flare (32.0%), thirst (16.8%), pruritus (11.6%), dry oral mucosa (14.0%), MS pain 9.6%), headache (8.0%), visual disturbances (4.0%), depression (4.0%), epistaxis (1.6%), impaired lipids (1.6%), impaired liver function test (1.2%), red eyes (0.8%), insomnia (0.85%), mild diarrhea (0.4%)
Gan (2013)^55^	Retrospective study (2b)	2255	22.5 / 71.4% M	Acne vulgaris: nodulocystic or severe (65.4%), moderate (11.9%), mild-moderate relapsing (22.7%)	NR	WBD: 0.5 mg/kg/d CD: Average 95.6 mg/kg Duration: Average 7.8 mo	33 (1.5%)	NR	Patient-reported	“Temporary”	NR	Cheilitis (64.8%), elevated lipids (12.2%), elevated liver function test (2.7%), muscle or joint pain (2.3%), headache (1.8%), mood change (1.6%), photosensitivity (1.5%), nausea (0.4%), blurred vision (0.4%), dizziness (0.2%)
Kmieć et al (2013)^56^	Prospective study (1b)	30	21.2 / 50% M	Papulopustular (n = 7), conglobate (n = 10) and phlegmonosa (n = 13) acne, no significant systemic disease	NR	WBD: 0.5-1.0 mg/kg/d CD:120-150 mg/kg Duration: 5-7 mo	Significant decrease in average total hair count (245.3 to 231.9, *P* < .05) density (336.61/cm to 326.01/cm, *P* < .05) and anagen hair proportion (73.0% to 71.4%, *P* < .05)	Exact time NR, measurements were performed at the end of treatment (5-7 mo)	FotoFinder dermatoscopy using TrichoScan Professional software (measured total hair count, density, proportion of anagen hair)	NR	NR	Skin dryness, pulling, burning, drying of mucous membranes, myalgia, headache, epistaxis (frequencies were NR)
Bray et al (2019)^57^	Prospective study (1b)	56	21 / 46.4% M	Acne vulgaris	NR	WBD: 1 mg/kg/d CD: 120 mg/kg Duration: 4 mo	1 (1.8%)	Exact time NR, noted at 2 and 4 mo F/U	Patient-reported	NR	One (1.8%) patient with dry skin, nosebleeds, lethargy and hair thinning discontinued isotretinoin use, however, not solely because of hair thinning	Significantly worsening mood, dry skin, nosebleeds, lethargy, blurred vision, sore eyes, polydipsia (frequencies were NR)
İslamoğlu and Altınyazar (2019)^11^	Prospective study (1b)	30	21.5 / 36.7% M	Severe acne vulgaris, no significant systemic disease	NR	WBD: 0.5 mg/kg/d CD: 45.6 mg/kg (calculated as 0.5 mg/kg/d × 91.25 d) Duration: 3 mo (91.25 d)	0 (0%)	N/A	FotoFinder dermatoscopy device using TrichoScan Professional program (measured total hair count, hair density, % of anagen and telogen hair, before and after treatment)	N/A	N/A	NR
Pandey and Agrawal (2019)^58^	Randomized, controlled comparative study (1b)	50	21.6 / 46% M	Moderate-to-severe acne, no significant systemic disease	NR	WBD: 0.5-0.6 mg/kg/d CD: 42.0-50.4 mg/kg (calculated as 0.5 mg/kg/d × 84 d to 0.6 mg/kg/d × 84 d) Duration: 3 mo (84 d)	0 (0%)	Exact time NR, noted at F/U every 4 wk for 12 wk	Patient-reported	NR	0 (0%)	Skin dryness (92%), nose dryness (27%), mouth dryness (100%), epistaxis (2%), face erythema (66%), scaling (14%), pruritus (50%), burning (12%), oiliness (6%), rash (8%), photosensitivity (12%)
		50	21.8 / 22% M	Moderate-to-severe acne, no significant systemic disease	Levocetrizine (5 mg)	WBD: 0.5-0.6 mg/kg/d CD: 42.0-50.4 mg/kg (calculated as 0.5 mg/kg/d × 84 d to 0.6 mg/kg/d × 84 d) Duration: 12 wk (84 d)	5 (10%)	Exact time NR, noted at F/U every 4 wk for 12 wk	Patient-reported	NR	0 (0%)	Skin dryness (76%), nose dryness (46%), mouth dryness (100%), eye dryness (22%), epistaxis (8%), face erythema (72%), scaling (16%), pruritus (18%), pruritus (18%), burning (4%), oiliness (25), rash (10%), photosensitivity (6%)
**Hair loss reported for pooled daily isotretinoin doses of <0.5mg/kg/d and ≥0.5mg/kg/d**†												
Rademaker (2010)^59^	Retrospective chart review (2b)	1743 (very low dose n = 450, low dose n = 471, medium n = 119, high n = 703)	Very low dose: 31.9 / 29.1% M Low dose: 20.9 / 47.2% M Medium dose: 20.5 / 53.8% M High dose: 19.7 / 58.3% M	Acne vulgaris (n = 1653), folliculitis (n = 38), rosacea or periorificial dermatitis (n = 68), seborrheic dermatitis (n = 50), others (n = 55). Some patients had >1 condition	NR	WBD:Very low: <0.25 mg/kg/d;low: 0.26-0.5 mg/kg/d; medium: 0.51-0.75 mg/kg/d;high: 0.76-1.0 mg/kg/d CD: 10-160 mg/kg (breakdown NR) Duration: 5-9 mo (breakdown for dose ranges NR)	2/1743 (reported hair loss was not broken down by isotretinoin dose)	NR	Patient-reported, hair loss was not asked about directly	NR	0 (0%) in all groups	Dose <0.50 mg/kg/d: cheilitis (63%), eczema (8%), tiredness (7%), mood change (5%), skin fragility (3%), epistaxis (3%), muscle ache (2%), eye problems (2%) Dose >0.5mg/kg/d: cheilitis (96%), eczema (16%), tiredness (18%), mood change (10%), skin fragility (9%), epistaxis (8%), muscle ache (6%), eye problems (5%) Dose <0.50mg/kg/d: none (34%), mild (59%), moderate (5%), severe (0.2%), stopped drug (1.4%) Dose >0.5mg/kg/d: none (1.6%), mild (82%), moderate (15%), severe (0.1%), stopped drug (1.3%) Other adverse effects (not broken down by dose): infections (2.6%), abnormal serum lipids (2.5%), slow response to treatment (2.2%), perifungal granulomas (2.1%), abnormal liver function tests (1.1%), sun sensitivity (1.0%), headache (0.7%), GI upset (0.3%), pregnancy (0.1%), acanthosis nigricans (0.1%), bed wetting (0.06%), bone calcification 0.06%), cardiomyopathy (0.06%), chilblains (0.1%), erythema nodosum (0.06%), facial erythema (0.06%), gallstones (1.0%), insomnia (0.06%), fainting (0.06%), IBD flare (1.0%), psoriasis flare (0.06%), gingival hyperplasia (1.0%), hematuria (0.06%), hyperthyroidism (0.06%), home sickness (0.06%), keratolysis exfoliate (0.06%), low platelets (0.06%), mouth ulcers (0.06%), polydipsia (0.06%), pterygium formation (0.06%), polycythemia (0.06%), tonsillitis (0.06%)
Demirseren et al (2017)^60^	Prospective study (1b)	300	22.2 / 33.7% M	Moderate and severe acne and unresponsive to systemic antibiotic and topical treatments: Papulopustular (63.4%), scar leaving comedogenic (27.9%), nodulocystic (8.7%)	NR	WBD : 0.25-1 mg/kg/d (study pooled patients in low (<0.5) and high (>0.5) doses in the analysis) CD: 120-150 mg/kg Hair loss occurrence CD median: 19.3 mg/kg Range: 2.3-87.0 mg/kg Duration: 2 mo	60 (20%)	Median: 4.0 wk Range: 1.0-24.0 wk	Patient-reported	NR	NR	Cheilitis (100%), dry face (61.3%), xerosis (57.0%), dry nose (55.0%), dermatitis (50.0%), epistaxis (46.7%), dry eye (43.3%), facial erythema (40.3%), acne flaring (38.0%), arthralgia (36.3%), fatigue (32.35), nervousness (24.3%), photosensitivity (24.0%), somnolence (21.3%), hypercholesterolemia (20.3%), myalgia (19.0%), palmar peeling (16.3%), headache (15.3%), weight gain (14.0%), hypertriglyceridemia (11.7%), herpes tip 1 (10.7%), menstrual irregularity (9.3%), weight loss (8.35), depressive symptoms (7.7%), hepatic dysfunction (7.3%), hand-feet sweating (5.7%), vaginal dryness (4.3%), ingrown toenail (4.3%), melasma (3.7%), increased CK (3.0%), photophobia (2.0%), nyctalopia (0.7%), conjunctivitis (0.7%), ear bleeding (0.3%)
Brzezinski et al (2017)^61^	Retrospective study (2b)	3525	18.5 / 47 % M	Moderate, severe, and nodulocystic inflammatory acne vulgaris	NR	WBD: 0.2-0.5 mg/kg/d CD: 56.6-141.4 mg/kg (calculated as 0.2 mg/kg/d × 282.88 d to 0.5 mg/kg/d × 282.88 d) Duration: Average 9.3 mo (282.88 d). Range 7-13 mo	154 (4.4%)	NR	Patient-reported	“Reported to persist even after therapy discontinuation”	1 (0.03%)	Dry lips (100%), xerosis (95.0%), facial erythema (66.2%), epistaxis (47.3%), cheilitis (41.8%), myalgias (38.8%), skin itching (38.1%), skin exfoliation (38.1%), tiredness (20.7%), headache (16.9%), joint ache (12.3%), retinoid dermatitis (11.7%), trachyonychia (10.4%), mood change (9.5%), dry eyes (5.7%), abdominal pain (3.7%) vision changes (2.9%), insomnia (2.8%), sun sensitivity (2.6%), dry dandruff (1.7%), dryness of mucous membranes of the mouth (1.4%), skin fragility (1.3%), polydipsia (1.0%), heavy menstrual periods (0.9%), insomnia (0.5%), herpes simplex (0.4%), paronychia (0.4%), Staphylococcus skin infections (0.2%), impetigo (0.2%), GI upset (0.2%), chalazion (0.1%), hyperhidrosis 0.1%), exacerbation cysts (0.06%), dry eye syndrome (0.06%), suicidal ideation (0.03%), intensification of fears (0.03%), blurred vision at night (0.03%), bleeding gums (0.03%), flare up of psoriasis (0.03%), herpes zoster (0.03)
Alshammari et al (2020)^62^	Cross-sectional/questionnaire (2b)	246	25.1 / 17% M	Acne vulgaris	NR	WBD: Not possible to calculate because weights were not reported. Fixed doses of 20-40 mg/d with the following BMI ranges: 130 patients with <25 kg/m^2^, 71 patients with 25-29.9 kg/m^2^, 26 patients with >30 kg/m^2^. CD: NR Duration: Average 7.2 mo	6 (2.4%)	NR	Patient-reported	NR	NR	None (49.2%), dry lips (3.7%), dry skin (3.7%), skin itching (3.3%), depressive symptoms (3.3%), joint and muscle pain (2.4%), increased heart rate (0.4%), headache (0.4%), impaired liver enzymes (0.4%)

*BMI*, Body mass index; *CD*, cumulative dose; *CK*, creatinine kinase; *CR*, case report; *CS*, case series; *F/U*, follow-up; *GI*, gastrointestinal; *IBD*, inflammatory bowel disease; *LDL*, low-density lipoprotein; *MS*, musculoskeletal; *N/A*, not applicable; *NR*, not reported; *WBD*, weight-based dose.

∗ This group of studies compared low versus high daily dosing within the articles and commented on hair loss at the respective doses.

† This group of studies did not clearly distinguish the side effects, including hair loss, in the low- versus high-dose isotretinoin groups. Although these data exist in the literature, they could not be analyzed further.

**Table II:** Compiled summary data of 17 studies (n = 3940) that clearly reported hair loss frequency in oral isotretinoin doses of <0.5 mg/kg/d and ≥0.5 mg/kg/d for acne patients∗

Daily isotretinoin dose	Mean age (y)	Number of men:women	Mean daily weight- based dose (mg/kg/d)	Mean duration of therapy (mo)	Mean cumulative dose (mg/kg)	Number of patients with hair loss	Total number of patients	Frequency of hair loss (%)
<0.5mg/kg/d	22.0	360:205	0.24†	4.4	70.6†	18	565	3.2
≥0.5mg/kg/d	22.4	2049:1326	0.59‡	6.5	97.2‡	192	3375	5.7

∗ The studies had to include clearly reported hair loss frequency for the low versus high dose of daily isotretinoin to be included in this summary table. Therefore, of the 22 reported studies, 17 were included in this summary table (studies by Kmieć et al,^56^ Rademaker et al,^18^ Demirseren et al,^60^ Alshammari et al,^62^ and Brzezinski et al^61^ were excluded).

† This value is the mean of 302 of the total 565 patients in this group. The entire low-dose group is not represented because 29 patients were on a fixed dose of 20 mg every 2 days and 234 patients were on a fixed dose of 20 mg/d. Although clearly low dose, weight was not provided to calculate the exact weight-based dose.

‡ This value is the mean of 3318 of the total 3375 patients in this group. The entire high-dose group is not represented because 57 patients were on an average fixed dose of 44.2 mg/d. Although clearly high dose, weight was not provided to calculate the exact weight-based dose.

### Evidence level

Of the 23 included studies, 16 were prospective trials, 3 were retrospective studies, and 3 were cross-sectional studies (Table I). Most of the studies were considered to have a relatively high (1b) level of evidence as per the Centre for Evidence-Based Medicine scale. The method of hair loss measurement varied, with 17 studies presenting patient self-reported hair loss, 3 studies reporting hair loss following an inquiry by a dermatologist, and 2 prospective studies measuring hair loss using a video dermatoscope.

### Dose-dependent hair loss frequency with isotretinoin

#### Isotretinoin dose of <0.5 mg/kg/d

A total of 565 acne patients were treated with <0.5 mg/kg/d of isotretinoin therapy (mean age, 22.0 years; men, 63.7%; Table II). The mean weight-based dose was 0.24 mg/kg/d, and the patients were treated for an average of 4.4 months to reach a mean cumulative dose of 70.6 mg/kg. Of the 565 patients in this group, the average weight-based and cumulative doses were calculated for 302 patients. Twenty-nine patients were on a fixed dose of 20 mg every 2 days, and 234 patients were on a fixed dose of 20 mg/d. These patients were not represented in the frequency analysis. Although this would be considered lower dose, exact weight-based dosing could not be calculated.

Overall, hair loss was reported in 18 (3.2%) patients of all patients treated with <0.5-mg/kg/d dosing (Table II).

#### Isotretinoin dose of ≥0.5 mg/kg/d

The group of acne patients treated with ≥0.5 mg/kg/d of isotretinoin therapy comprised 3375 patients (mean age, 22.4 years; men, 60.7%; Table II). The mean weight-based dose was 0.59 mg/kg/d, and the patients were treated for an average of 6.5 months to reach a mean cumulative dose of 97.2 mg/kg. Of the 3375 patients, the average weight-based and cumulative doses were calculated for 3318 patients. The entire high-dose group was not represented because 57 patients were on an average fixed dose of 44.2 mg/d.

Overall, hair loss was reported in 192 (5.7%) patients of all patients treated with ≥0.5 mg/kg/d of isotretinoin (Table II).

### Time of hair loss onset with isotretinoin

The timing of hair loss onset during isotretinoin dose escalation has only been documented in 1 study to date. Demirseren et al^60^ found that 20% of 300 patients experienced hair loss, with a median time of onset at 4 weeks and a wide range of 1 to 24 weeks.

Two prospective studies quantified hair loss with FotoFinder dermatoscope using TrichoScan Professional software.^11^^,^^56^ The first study of 30 patients reported no significant change in the total hair count, hair density, or percentage of telogen hair within 3 months of 0.5-mg/kg/d isotretinoin treatment.^11^ The second study treated 30 acne patients with higher isotretinoin doses (0.5-1 mg/kg/d) for a longer duration (4-7 months) and described a significant decrease in the total hair count, hair density, and percentage of anagen hair.^56^ It is unclear whether it was the increased daily dosing or the higher cumulative dose with the longer treatment duration that contributed to the increased hair loss, but it does suggest that one of these factors contributed to telogen effluvium, especially when these data are compared with the first FotoFinder study described earlier.

### Reversibility of hair loss with isotretinoin

Of the 22 studies reviewed, only 2 discussed the reversibility of hair loss. Brzezinski et al^61^ described hair loss in 154 patients on isotretinoin and commented on its persistence after completing treatment; however, there were no data to support this comment, and the time frame as well as the number of patients affected were unclear. Conversely, a retrospective study by Gan et al^55^ reported that hair loss was temporary in 33 patients who experienced hair loss on high-dose isotretinoin.

## Discussion

A summary of the available data suggested that patients on <0.5 mg/kg/d of isotretinoin experienced hair loss at a frequency of 3.2% versus those on ≥0.5 mg/kg/d, who experienced hair loss at a frequency of 5.7%. Inferential statistics comparing the groups was not possible because of the heterogeneity of the data.

### Hair loss onset and prognosis

Drug-induced telogen hair loss has previously been reported to start after 12 weeks of therapy.^63^ The drug prematurely transitions the follicles from the anagen phase to the telogen phase, which has a duration of 3 months prior to shedding.^64^^,^^65^ Data on hair loss with isotretinoin dose escalation are lacking and have only been documented in a small cohort of patients, with a median time of onset at 4 weeks.^60^ It is unclear whether the duration of treatment or the cumulative isotretinoin dose plays a significant role in the timing of hair loss onset, but contrasting results of 2 similar prospective studies using FotoFinder trichoscopy at doses of 0.5 and ≥0.5 mg/kg suggested that higher doses for longer periods (>4 months) result in higher rates of telogen effluvium.

The reversibility and extent of hair regrowth are important to consider because patients may view permanent hair thinning as a barrier to therapy. Although the product monographs of isotretinoin formulations (Clarus, Epuris, and Accutane) warn that hair loss may persist after treatment is completed, there is no definitive evidence to support this prognosis.^55^^,^^61^

### Impact on clinical practice

Hair loss has been described as a rare side effect of isotretinoin in product monographs and in the literature.^13^, ^14^, ^15^ This systematic review demonstrated a 3.2% to 5.7% frequency of hair loss, which is comparable with the frequency of the side effect of dry eyes (5.7% frequency).^61^ This highlights the importance of counseling and monitoring for hair loss during isotretinoin treatment. The most up-to-date recommendations for isotretinoin therapy suggest that the patient should remain clear of acne for 1 to 2 months prior to discontinuing the treatment in order to reduce recurrence rates.^5^^,^^37^^,^^39^ These suggestions are based on a systematic literature review by Tan et al,^39^ which showed that daily and cumulative doses did not influence the relapse rates as long as the treatment was continued for >2 months after acne resolution. Therefore, in patients concerned about hair loss, dermatologists may consider prescribing lower daily isotretinoin doses over a longer period of time to reach the same goal prior to discontinuation. This approach has been successful, with other reported side effects.

Although data specific to hair loss have not been reported, previous studies have shown that low daily doses in the range of 0.10 to 0.40 mg/kg can be efficacious while reducing the risk of mucocutaneous side effects.^23^^,^^29^^,^^43^ Intermittent dosing may also represent an alternative treatment, especially for mild-to-moderate acne. Daily isotretinoin for 1 week every 4 weeks over a duration of 6 months was shown to be an effective treatment for mild-to-moderate acne and resulted in minimal side effects.^66^^,^^67^ For patients with more severe acne, there were fewer side effects when isotretinoin was prescribed for either the first 10 days of each month for 6 months (a cumulative dose of 25 mg/kg) or each day in the first month and the first 10 days of each subsequent month for 5 months (a cumulative dose of 49 mg/kg) at 0.5 mg/kg/d.^49^ If hair thinning is a serious concern and a potential barrier to the systemic management of acne vulgaris, one of these low-dose regimens might be an alternative approach, although definitive data for hair loss are lacking.

### Study limitations

There are limitations resulting from the available data in the literature. Because of the study design, heterogeneity, and large difference in the number of patients between the groups, a statistical analysis comparing the <0.5-mg/kg/d and ≥0.5-mg/kg/d isotretinoin groups was not possible. Most of the included studies were not randomized and, thus, limited the comparison of adverse effects between the isotretinoin doses. Moreover, there were not enough data available to make comparisons of hair loss with isotretinoin use in men versus that with isotretinoin use in women. Similarly, data on confounding variables, such as iron deficiency anemia, were not available. Despite the large volume of literature on isotretinoin, there are a limited number of studies that assessed hair loss (n = 22, Fig 1), of which only 6 studies reported hair loss outcomes in patients using <0.5 mg/kg/d. Furthermore, the majority of the outcomes were patient-reported and, thus, prone to subjectivity. Although the studies that reported hair loss had a lower mean proportional cumulative dose (70.6-97.6 mg/kg, Table II) compared with the frequently targeted cumulative dose of 120 to 150 mg/kg, newer recommendations suggest that maintained clearance is a more important therapeutic target than cumulative dose.^19^^,^^20^^,^^37^ The relationship between cumulative dose and hair loss could not be elucidated from the data available.

Despite these limitations, this systematic review comprehensively summarized the literature to date, identified the gaps in the literature, and demonstrated evidence for the frequency of isotretinoin-induced hair loss (up to 5.7%). Given the volume of patients with acne on isotretinoin in North America, dose reduction could be further explored as an alternative to discontinuation for patients experiencing hair loss. A high-quality prospective study is required to formally assess the dose dependency and impact of a cumulative dose.

## Conclusion

Physician knowledge of the frequency, timing, dose dependency, and reversibility of hair loss with isotretinoin treatment is limited because of the lack of discussion in the literature. This systematic review analyzed the data available and suggested that <0.5-mg/kg/day isotretinoin dosing results in hair loss at a frequency of 3.2%, whereas ≥0.5-mg/kg/day isotretinoin dosing demonstrates hair loss at a frequency of 5.7%. This frequency is not dissimilar to many of the side effects commonly discussed with patients and impacts a large patient population because of the frequency of isotretinoin prescription.^10^ The role of dose reduction in hair loss frequency is important to establish because it would allow patients with acne and distressing levels of hair loss to still receive effective therapy.

## Conflicts of interest

None disclosed.
